# Cause of seizures: Lungs, not the brain

**DOI:** 10.4103/0970-2113.76306

**Published:** 2011

**Authors:** Amitesh Aggarwal, Vishal Sharma, Surendra Rajpal, Shridhar Dwivedi

**Affiliations:** *University College of Medical Sciences and GTB Hospital, New Delhi, India*

**Keywords:** Hyponatremia, pneumonia, SIADH

## Abstract

Hyponatremia is a common electrolyte abnormality but is usually asymptomatic and is often neglected. We present case of a 22-year-old diabetic male who presented with generalized tonic clonic seizures which was later found to be a consequence of hyponatremia. Further investigation unravels the presence of SIADH which was eventually found to be due to the consolidation of the left lingual lobe of lung. This case emphasizes the need for a thorough workup to identify the etiology of hyponatremia as it may unmask a treatable entity.

## INTRODUCTION

Hyponatremia is the commonest electrolyte abnormality seen in clinical settings but is often a neglected entity.[1] We present case of a 22-year-old diabetic male who presented with generalized tonic clonic seizures which was later found to be a consequence of hyponatremia. Further investigation unravels interesting findings emphasizing the need for a thorough workup to identify the underlying treatable entity.

## CASE REPORT

A 22-year-old male presented with a history of generalized tonic clonic seizures (GTCS). His mother reported the occurrence of two previous episodes of GTCS in past 15 days. He had no family history of seizures. He was a known type 1 diabetic for past 3 years on regular insulin therapy. Six months back he had finished a 6-month course of antitubercular therapy. His father had died of pulmonary tuberculosis 8 years back. Rest of the family history was unremarkable. At admission, the patient was comatose but his vitals were stable. He was afebrile, and had no meningeal signs or focal neurological deficit.

**Figure 1:**
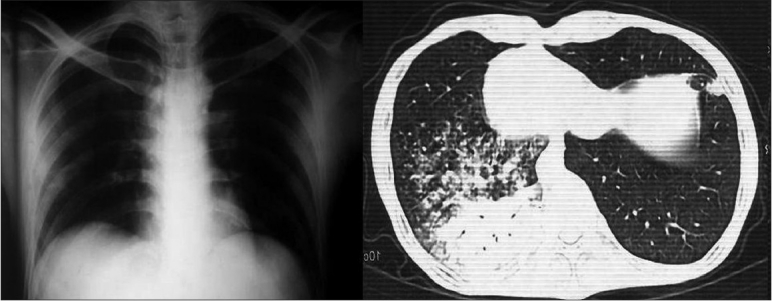
Normal chest x-ray but CECT revealing consolidation

At the time of first seizure, he had elevated blood glucose without ketoacidosis. His chest roentgenogram and brain CECT were normal and the metabolic profile was unremarkable except for a low serum sodium level (104 meq/L) at that time. The patient was initially administered the loading dose of phenytoin. His hemogram and metabolic profile were normal except for low serum sodium (116 meq/L). Rest of the workup for the cause of seizures was unremarkable. A possibility of hyponatremia as the cause of seizure was kept. He was administered hypertonic saline. The patient improved when serum sodium was corrected to 125 meq/L over 24 h. His calculated plasma osmolality at presentation was 254 mosm/L. His urine sodium was 74 meq/L and urine specific gravity was 1.015. His serum uric acid was 2 mg/dL. The patient was clinically euvolemic. Workup for hyponatremia including complete blood count, liver and renal function tests, serum proteins, lipid profile, urine examination, serum cortisol, thyroid profile, renal ultrasonography, HIV, contrast-enhanced MRI of brain, and CSF examination (including ADA, HSV1, and two antigens) were normal. EEG was not done as the patient improved with the correction of hyponatremia. In view of this, the patient was suspected to have SIADH. He was not taking any drugs known to cause SIADH. Repeated chest X-ray was normal. In view of the past history of pulmonary tuberculosis and presence of SIADH, a chest CECT was done which revealed evidence of the collapse and consolidation in the lung [Figure 1]. The patient was put on broad-spectrum antibiotics, and on follow-up, the patient remained asymptomatic and maintained normal serum sodium levels.

## DISCUSSION

Hyponatremia is a common electrolyte abnormality but is usually asymptomatic. Mortality rates do not differ between patients with symptomatic or asymptomatic hyponatremia.[1] In the present case, we had a patient who presented due to seizures resulting from hyponatremia. The euvolemic hyponatremia, low plasma osmolality, and high urinary sodium in spite of hyponatremia and hypouricemia suggested the presence of SIADH.[2] We had no clinical clue to the actual cause of SIADH. His chest radiographs and MRI of brain were normal. The patient was finally found to have a consolidation of the left lingual lobe of lung. Most cases of hyponatremia in a clinical setting result from SIADH. The search for the etiology of SIADH should exclude the possibility of underlying neoplasm, pulmonary diseases, CNS disorders, drugs, AIDS, etc.[Table 1]

**Table 1:** Causes of SIADH[3,4]

Neoplastic	Carcinoma lung, gastrointestinal neoplasms, ovarian carcinoma, thymoma, etc.
Neurologic disorders	Head trauma, encephalitis, meningitis, cerebrovascular occlusions, hemorrhage, cavernous sinus thrombosis, Guillain–Barré syndrome, multiple sclerosis, hydrocephalus, psychosis, peripheral neuropathy, congenital malformations like agenesis of the corpus callosum, cleft lip/palate, etc
Pulmonary diseases	Pneumonia, lung abscess, cavitation (aspergillosis), tuberculosis, carcinoma
Drugs	Vasopressin or desmopressin, chlorpropamide, vincristine, carbamazepine, nicotine, phenothiazines, cyclophosphamide, tricyclic antidepressants, monoamine oxidase inhibitors, serotonin reuptake inhibitors
Others	Infection (Legionella, HIV), metabolic (acute intermittent porphyria)

Treatment of hyponatremia depends on whether it has developed acutely or if it is chronic. Acute severe hyponatremia associated with CNS manifestations including seizures should be corrected by the infusion of hypertonic saline to target a rate of increase in plasma sodium by around 1 meq/L/h and not more than 12 meq/L in 24 h. This is to ensure that the osmotic demyelination syndrome associated with the rapid correction of hyponatremia is avoided. In persence of less severe symptoms, restriction of fluid intake and administration of loop diuretics may be used. Chronic SIADH may warrant therapy with demeclocycline or fludrocortisone.[3,4] However, newly discovered vasopressin antagonists (conivapton, lixivaptan, tolvaptan) have been found to increase free water excretion and therefore offer further advantages as they do not alter sodium and potassium excretion.[2]

All in all, hyponatremia remains an often neglected entity due to usually asymptomatic nature. This case emphasizes the need for a thorough workup to identify the aetiology of hyponatremia as it may unmask a treatable entity.

## References

[1] Berl T, Verbalis J, Brenner BM, Rector FC (2004). Pathophysiology of water metabolism. Brenner and Rector’s the kidney.

[2] Siragy HM (2006). Hyponatremia, fluid-electrolyte disorders, and the syndrome of inappropriate antidiuretic hormone secretion: Diagnosis and treatment options. Endocr Pract.

[3] Singer GG, Brenner BM, Fauci AS, Braunwald E, Kasper DL, Hauser SL, Longo DL, Jameson JL (2008). Fluid and electrolyte disturbances. Harrison’s principles of internal medicine.

[4] Robertson GL, Fauci AS, Braunwald E, Kasper DL, Hauser SL, Longo DL, Jameson JL (2008). Disorders of the neurohypophysis. Harrison–s principles of internal medicine.

